# Cultural Effects on the Performance of Older Haitian Immigrants on Timed Cognitive Tests

**DOI:** 10.18103/mra.v12i11.5868

**Published:** 2024-11-30

**Authors:** Marie Adonis-Rizzo, Ruth M. Tappen, Monica Rosselli, David Newman, Joshua Conniff, Jinwoo Jang, KwangSoo Yang, Borko Furht

**Affiliations:** 1 Christine E. Lynn College of Nursing, Florida Atlantic University, 777 Glades Road NU-84, Boca Raton, FL 33431, USA; 2 Department of Psychology, Florida Atlantic University, 3200 College Ave, ES-BC52, Room 268, Davie, FL, 33314, USA; 3 Neuropsychology Lab, Department of Psychology, Florida Atlantic University, 777 Glades Road, Boca Raton, FL, 33431, USA; 4 Department of Civil Engineering and I-Sense, Florida Atlantic University, 777 Glades Road, EE 503D, Boca Raton, FL, 33431; 5 Department of Electrical Engineering and Computer Science, Florida Atlantic University, 777 Glades Road, EE 428 & 526, Boca Raton, FL, 33431

## Abstract

**Background:**

Ignoring the cultural factors that can affect performance on cognitive tests may result in use of tests that have not been validated for that group. One example is testing of Haitian Creole speaking adults who are increasingly affected by Alzheimer’s disease and related dementias, for whom few tests have been validated.

**Aims:**

Our purpose is to describe differences in timed test performance between Haitian Creole and English-speaking participants and explore factors that may account for any differences in results found.

**Methods:**

Data was obtained from an ongoing longitudinal driving and cognition study “In Vehicle Sensors to Detect Cognitive Change in Older Drivers.” Two groups consisting of 12 Creole speaking and 12 English speaking older adults were matched by age and gender. Test scores were selected from the battery of tests administered in the parent study. The measures were translated by two bilingual Creole-English researchers. Group performance on five timed cognitive tests commonly used in research was compared.

**Results:**

The English-speaking group’s mean scores were significantly higher than the Creole speaking group on the MoCA and the timed Animal category fluency, letter P fluency, Stroop Color Test, and Trail Making Test A and B. The most significant effects were noted in Letter P fluency, Trail Making Test A and B and Animal category fluency where the differences had large effect sizes. However, the Creole speaking group had higher mean scores than the English-Speaking group on the Stroop Color Word Test, although the difference was not statistically significant. It was not feasible to match education levels due to the differences in years of education across the groups. These results highlight the significant role of culture and linguistic context in cognitive task performance.

**Conclusions:**

The results suggest performance in cognitive testing among non-English speaking groups may be impacted by cultural factors related to time perception and the testing approach employed, leading to misinterpretation and misdiagnosis. Future studies should explore the fairness of various cognitive testing approaches with Haitian older adults and other societies with cultures and educational approaches different from those of Western cultures.

## Introduction

Time constraints on neurocognitive tests may impact performance for many older adults, particularly those from societies where emphasis is placed upon accuracy rather than speed, potentially contributing to misdiagnosis of Alzheimer’s disease (AD) in many older members of ethnic minority groups^1^. In 2024, 6.9 million older adults in the US and seven million in Europe were living with AD or related dementia. An estimated fifty-five million people globally have been diagnosed with AD or related dementia^2^. Underrepresented groups, such as Black and Hispanic populations, are reported to experience higher rates of the disease which are attributed to factors such as low socioeconomic status and the prevalence of atherosclerotic disease but may also be due in part to the absence of test norms for these groups^2^. The World Health Organization reported that 955 or 1.9 % of total deaths in Haiti are due to Alzheimer’s disease and dementia^3^.

Early screening and diagnosis are crucial for timely treatment and slowing the disease’s progression. However, there are concerns about accurately screening and diagnosing individuals from culturally, educationally and linguistically diverse backgrounds.

Recent efforts in cross-cultural research on cognitive testing reflect recognition of the need to evaluate members of ethnic minority groups globally^4^. Caution is needed when using normative data from other groups, as it was neither intended nor developed for them. More specifically, there is also a tendency to group all Black individuals together in research studies instead of recognizing the cultural and educational differences among recent immigrants from Africa and the Caribbean vs those whose families may have been resident in the US, UK or other countries for several generations^4,5,6^. South Florida is home to a large and growing population of Afro-Caribbeans, including Haitian Americans. Similarly, large groups of Haitians have established communities in France, Canada, and French Caribbean territories. To date, there are no validated or normed cognitive tests specifically designed for this group of older adults, who are culturally and linguistically distinct. Literature on cognitive testing in this population is also very scarce. The cognitive tests currently available were designed for Western countries and English speakers ^7,5^. Some norms have been established for African Americans on tests such as the MoCA, Boston Naming Test, Controlled Word Association, Category Fluency, Animal Naming, Token Test, WRAT-3 Reading, Trail Making Test, Stroop Test, and Judgment of Line Orientation ^7,5^. There is no evidence, however, of their applicability or fairness when administered to individuals of Caribbean or African origin.

Several researchers have identified cultural factors such as time orientation as potential contributors to poor performance on timed neuropsychological tests^8,9^. Cognitive tests that allow a limited amount of time may be influenced by familiarity with the testing items and the respondent’s culture-based attitudes toward time. Ardila et al. (1989)^10^ argue that the quality of education also impacts cognitive abilities, particularly in ethnic minority groups, who may have experienced educational disparities, particularly related to quality of instruction.

A body of literature on the test performance of non-English speakers has shown mixed findings. Rosselli et al. (2000)^11^ found that bilingual Spanish-English individuals performed similarly to native English speakers on verbal fluency and repetition tests administered in English. Other studies suggest that bilinguals, through acquiring language skills and vocabulary in a new language, may perform better in executive function tasks including inhibitory control and cognitive flexibility^12^. Kisser et al. (2012)^13^ corroborated these findings in their study, where they administered selected measures to native and non-native English speakers. They found that non-native English speakers performed worse on language processing tasks, letter fluency, category fluency, the Cognitive Estimation Test, and Trail Making Test A. However, they had no significant differences in tests of executive functioning.

Other researchers have suggested that slower language processing in non-English speakers may play a role in poorer performance on letter and category fluency tasks, as more time is required for word generation^14^. However, language interference alone may not fully explain these differences. In a European study, for example, Finnish and Scandinavian participants demonstrated slower processing and working memory speeds than did American, French, and German participants^15^. Similarly, Eizaguire et. al. (2020)^16^ found that the performance of Argentinian and Mexican groups were similar on the oral processing speed task, but scores were lower than the Anglo-American groups. These differences were attributed at least in part to cultural attitudes toward test-taking, where accuracy is valued over speed. Rosselli and Ardila (2003)^17^ found that Americans tend to focus on fast-paced performance and may do better on cognitive tasks requiring speed than do members of other cultures that emphasize accuracy over speed.

Cognitive test performance depends upon education level and the quality of the education obtained^8^. Socioeconomic and political factors and forced migration have contributed to the low literacy rate of Haitian immigrants. Approximately 8% of Haitian schools are public and 92 % are private, resulting in a lack of access to school for many families living in rural areas and neighboring towns^18^. Creole is the primary spoken language in Haiti, but French is the primary written language in schools and on the national exams that are required to pass grades 6, 9, and 13, leading to premature dropout from school. Haitian immigrants residing in the US, Canada, Europe, and the Caribbean may not have been exposed to or acquired the skills and strategies needed to perform well on timed cognitive tests.

The purpose of the current study was to explore differences in neuropsychological test performance of two groups of older adults, Creole-speaking immigrants to the U.S. educated primarily in Haiti and English-speaking individuals born and educated in the U.S. We describe the differences in test performance between the two groups and explore the factors that may account for the differences found. It was anticipated that the English-speaking group’s mean scores would be higher on the timed tests due to factors such as differences in education, cultural differences in time perception and lack of familiarity with testing of this type on the part of the Haitian Creole speakers.

## Method

### DESIGN

This is an exploratory comparative matched pairs design study conducted on data from an ongoing study. This design allowed for the comparison of mean scores on cognitive timed tests of two groups, native Haitian Creole speakers and native English Speakers^19,20,21^.

The two groups consisting of twelve native Haitian Creole speaking and twelve native English-speaking older adults matched by age and gender were selected from an ongoing longitudinal driving and cognition study “In Vehicle Sensors to Detect Cognitive Change in Older Drivers^22^.” The diverse sample of older adults recruited from Southeast Florida includes African American, Afro-Caribbean Hispanic and non-Hispanic European Americans. The participants signed consents in their primary language. The study from which this data was obtained was approved by Florida Atlantic University IRB.

### MEASURES

A battery of cognitive tests was administered to participants in the parent study to detect changes that may have occurred over time. Before use, two bilingual native Creole-English researchers translated and adapted the measures to Haitian Creole. Several meetings with two researchers in neuropsychology took place before and during the translation process to ensure the retention of the meaning of test questions. Since there are no validated Haitian Creole cognitive tests, we sought feedback from an older native Haitian Creole speaker on the translated items, understanding that Haitian Creole has dialects that vary geographically^23^. For this analysis, we compared the groups’ performance on five timed cognitive tests used in traditional neuropsychological assessment.

### GLOBAL COGNITIVE FUNCTION

The Montreal Cognitive Assessment (MoCA) is frequently used as a screening test. The MoCA assesses short-term memory, visuospatial function, executive function, attention, concentration, working memory, language, and orientation^24^, and takes about 10 minutes to administer. The whole test is not timed, but the verbal fluency item (Letter F) is timed. The participant is asked to cite as many words that begin with the letter F as possible in 1 minute.

### EXECUTIVE FUNCTION

The Trail Making Test (TMT), which consists of two parts, TMT A and TMT B, is a measure of executive function, cognitive response, and shifting attention^25,26^ Errors made and the time to complete the test in seconds are recorded. TMT A involves connecting twenty-five scattered numbers in numerical order, allowing a maximum of 150 seconds to complete. TMT B consists of twenty-five alternating numbers and letters to be connected in numerical and alphabetical order allowing a maximum of 300 seconds to complete.

The Stroop-Color Word Test (SCWT) is used to evaluate the ability to inhibit cognitive interference, which may occur while processing two stimuli at the same time^27^ The participant has 45 seconds to complete reading color words or naming colors. The SCWT has three conditions. The first condition (W) requires participants to read a list of words (names of colors printed in black ink) as quickly as possible. The color condition requires participants to name colors (C) from Xs printed in three different colors), and the color-word (CW) condition requires the participants to name the color of the ink of color names written in different ink colors. The Stroop effect is when it’s more difficult and takes more time to name the color of the ink when the color name is written in a different ink because there is an automatic tendency to read the word and, therefore, to perform well, the participant must inhibit the reading of the word. Participants were evaluated for color blindness prior to administering the test. The colors used were red, green, and blue. The length of the color words differed in Creole and English. Only the word red was longer in Creole (Rouj)^28^. Green and Blue were shorter in Creole (Vet and Ble)^28^.

### LANGUAGE

Verbal Fluency was assessed using category (animals) and phonemic (letter P) items. This test also measures executive function and language processing^29.^ The participant is asked to list as many animals or words as they can in one minute.

The participants were evaluated by trained psychometricians. A bilingual Haitian Creole-English speaker tested the Creole participants (M. A-R).

### TESTING LOCATIONS

English speaking participants were evaluated on several campuses of Florida Atlantic University in Broward and Palm Beach Counties. All but two of the Haitian Creole speaking participants were evaluated by their choice at secondary sites including their place of worship.

Data collection was scheduled every three months in this longitudinal study following two baseline testing sessions, divided to reduce participant fatigue. Baseline test results were used in this analysis. Demographic information was obtained at visit 1. The MoCA, Trail Making Test A and B, Animal category, letter P fluency, and Stroop Color Test were administered at Baseline visit 2.

### STATISTICAL ANALYSIS

Statistical analysis was conducted using SPSS^30^. One-tailed independent samples *t*-tests were conducted to examine whether the results of the cognitive timed measures were different across the 12 Haitian Creole speakers and 12 English speakers.

## Results

### SAMPLE CHARACTERISTICS

The groups were closely matched by gender with 50% male and 50% female in each group and by age ((*t*(22) = 0.17, *p*= .431). See Table 1.

We made considerable effort to match the groups by education level, but it was not feasible because the mean years of education were significantly different between the Haitian Creole and English speakers, (t (13.55) = −2.36, *p =* .034). See Table 2

### COGNITIVE TEST ANALYSES

The two groups’ means and standard deviations for different cognitive tests are presented in Table 3. Significant differences were found between the groups in the performance of all tests except the Color-Word interference. The MoCA total adjusted scores for Haitian Creole speakers differed significantly from those of the English speakers. The mean for Haitian Creole speakers was significantly lower than the mean for English speakers, with a medium effect size. Likewise, there were significant differences found in letter P words generated for the Haitian Creole speaking group versus the English-speaking group with a large effect size. However, the mean of Letter_P_rule_violations was not significantly different between the Haitian Creole speaking and English-speaking group. English speakers produced a significantly higher total number of animals correctly named than did the Creole speaking group with a large effect size. There were no significant differences in rule violations between the two groups. See Table 3.

Haitian Creole speaking participants were slower than the English-speaking group in completing the TMT A, with a large effect size. Similarly, the mean TMT B time in seconds was significantly different between the two groups. It should be noted that at least 5 Creole speaking participants, including those with no formal education, were unable to complete TMT B. When this occurs, a maximum of 300 seconds is then recorded. See Table 3.

Analyses of the two Stroop tests results revealed a significant difference in score between the groups, also with a large effect size. The mean reading total for the Creole speaking group was lower than that of the English speaking group, as was the Name Color total for which the mean was significantly lower for the Creole speaking participants (M= 44.8; SD = 16.9) as compared with the English speaking group However, the Creole speaking group performed better on the color word total than did the English speaking group. Although this difference was not statistically significant, the moderate effect was noteworthy.

## Discussion

This study explored the challenges Haitian Creole speaking older adults face when subjected to neuropsychological assessments validated for English-speaking populations with higher levels of education. Our research shows a significant difference in test performance between Haitian Creole speakers and their English-speaking counterparts on timed neuropsychological measures when matched on age and gender. Bugallo-Carrera et al. (2024)^31^ and Malek-Ahmadi et al. (2015)^32^ reported that demographic data such as age and sex have a significant impact on MoCA test results. Our groups were equivalent in their mean age and gender distribution. The results from our Haitian Creole speaking group highlight the critical role of cultural and linguistic context in cognitive task performance. The differences between groups in level of education is also a factor in determining test outcomes, but we cannot rule out the effect of the cultural context in the interpretation of cognitive outcomes^17^.The observation that initiation time (the pause to think about one’s answers before beginning to list words that start with a particular letter, for example) in cognitive tasks often causes a delay among participants echoes previous research with underrepresented ethnic/-racial groups^9,33^

The parent study utilized the MoCA to screen potential participants for eligibility^22.^ Despite its widespread translation and validation in approximately 100 languages, it has not been validated in Haitian Creole, an oversight in achieving linguistic and cultural representation in cognitive assessment. The suggested cutoff scores for identifying mild cognitive impairment (MCI) in various racial and ethnic groups with low levels of education reflect a recognition of the importance of avoiding misdiagnosis, especially among ethnically underrepresented groups such as Haitian Americans. Given our findings of a mean MoCA score of 23.1 among the Haitian Creole speaking group compared to 25.7 in the English-speaking group, applying existing norms would invariably result in exclusion of eligible participants. Previous authors have suggested the importance of better understanding the effects of the level of education on the MoCA and the need to create appropriate cutoff scores for older adults with very low education^34,35,36^. Moreover our results support previous findings suggesting that linguistic and cultural factors must be considered when the MoCA is used as a clinical screening test of individuals with limited education due to the high number of items that depend on culture and academic skills such as reading, writing and math^37^.

In the verbal fluency tests, the English-speaking group generated significantly more words than did the Haitian Creole-speaking group. This is consistent with previous studies on verbal fluency tasks across languages^38^. Production of words on a verbal fluency test is affected by word frequency^39^ and the frequency of words that start with a particular letter or that belong to a particular semantic category varies across languages.

Stewart et al. (2001)^6^ found that Afro Caribbean participants aged 55–75 scored lower on verbal fluency and the Boston Naming Test, while their memory test scores were similar. The Afro-Caribbean population is not a monolithic group, however, so caution should still be taken with assigning norms from one ethnic group or one region to the next. Stewart et al, (2001)^6^ for example, did not specify the ethnicities or preferred language of their groups, highlighting the challenges of cognitive screening in underrepresented populations. The score difference may be because of speed emphasis in timed tests, language processing, age, and education^40^. This study explored the possible effect of timed testing and speed on text performance.

There were also no significant differences in rule violations between the groups. Instead, the most noticeable differences were observed in the total scores and the number of words and animals generated. It was observed (by M. A-R) that a delayed response time occurred in the Creole speaking group during testing, with a long pause before initiating the first word. After stopping the clock, participants often provided additional words and animals, another example of focusing on accuracy rather than speed. Information processing may also be affected by bilingualism as was suggested by the Creole speaking group’s higher Stroop Color Word subtest. This study did not address the impact of acculturation on verbal fluency test performance. The Creole-speaking participants had lived in the US for over 15 years, and all but four of them had received some formal schooling in the US. Five participants were Creole-English bilingual while seven participants were Creole monolingual. A study conducted by Manly et al. (2004)^33^ observed that the correlation between reading level and test performance was primarily in measures of verbal abstraction, naming, and phonemic fluency. However, when other demographic factors such as acculturation and years of education were considered alongside reading level, the influence on test performance was attenuated.

The Haitian Creole speaking groups clearly took longer to complete the TMT A and TMT B tests than did the English group. The effect sizes were large. In this study, we cannot definitively conclude that a combination of age and education level were responsible for the lower scores in the Creole speaking group as we were unable to match participants on years of education. We do suggest, however, that education level as well as type of education may have played a role. Ardilla et al., (2005)^8^ noted that lack of exposure to specific tasks in a cognitive test would impact the test performance. Five members of the Creole speaking group did not complete the TMT B test within the 300 seconds allowed. A few of them became frustrated and asked permission to stop doing the task. The ones who completed both trials were still slower than the English groups and took the total time allocated for each test, 150 for TMT A and 300 for TMT B. Results for this study support previous findings suggesting that the TMT has an educational bias that makes it inapplicable to people with low levels of education due to its dependence on the alphabet^41^.

Further investigation is warranted regarding task performance beyond the designated time. Agranovich et al. (2011)^9^ examined cultural differences in time attitudes and their effect on timed neuropsychological tests. After administering several neuropsychological tests, their participants completed the Culture of Time Inventory-33 Items (COTI-33). They reported that Americans outperformed Russians on several subsets of items and that attitudes towards time affected test results.

Haitian-Creole speaking participants were slower in the Stroop reading total number test and Stroop naming color total number than the English-speaking group. However, the Creole speaking group scored better on the color name total than did the English-speaking group. This difference was marginally significant, and therefore, a larger sample should be compared to corroborate this finding. One explanation of this preliminary finding is that the lower level of reading of the Haitian Creole group compared to the English-speaking group may not create the interference that is expected in this part of the test. This result questions the validity of the Stroop test of inhibitory control test in individuals with low levels of education. Although we are not certain why they were faster in the latter task, this is consistent with previous research on bilingual participants. Bilingual and monolinguals experience different Stroop interference and facilitation effects, revealing a bilingual cognitive advantage^42^. Future research with a larger sample should be done to corroborate this finding.

## Conclusion

In this study we found differences in performance on timed cognitive tests between Haitian Creole-speaking and US English-Speaking older adults and explored some of the factors that may account for these differences. Since the groups were well matched in age and gender, we can begin to understand how early educational experiences have a greater impact on test performance than is usually acknowledged. Culture specific factors such as time perception and emphasis on accuracy vs. speed in test taking may play a vital role in poorer performance. We had a mixed group of older Haitian Creole speaking adults with varying levels of literacy. While the majority had less than twelve years of education, two males attended college in the US, two males attended trade school in the US, and three females completed nursing assistant programs. Separate norms and assessment profiles for ethnic groups such as Haitian older adults are needed. Future studies of cognitive tests of various domains that address differences in literacy level, linguistic abilities and educational emphases in testing may explain the differences in performance, help guide cognitive test development and prevent inappropriate labeling of individuals as having dementia. With the growing population of disadvantaged minority groups across the US, Europe and other regions, and their reported increasing rates of Alzheimer’s disease and related dementias, accurate screening for early diagnosis is needed.

## Figures and Tables

**Table 1.** Demographic Profiles of Creole and English Samples on Matching Variables

	**Creole (N=12)**		**English (N=12)**		
**Variable**	*M*	*SD*	*M*	*SD*	*P*
Age	74.0	5.7	73.6	6.0	.431
Education	10.3	6.1	14.7	2.1	.034

**Table 2.** One -Tailed Independent Samples t-Test for education by Group

	Creole			English					
Variable	*M*	*SD*	*n*	*M*	*SD*	*n*	*t*	*P*	*d*
education	10.25	6.14	12	14.67	2.1	12	2.36	0.34	.96

*Note:* N = 24. Degrees of Freedom for the *t*-statistic = 13.55. *d* represents Cohen’s *d.*

**Table 3.** Timed Cognitive Test Results for the Creole and English-speaking groups

Test	Creole			English							
	*M*	*SD*	*n*	*M*	*SD*	*n*	*t*	*df*	*P*	*SE*	*d*
MoCA	23.1	2.7	12	25.7	1.6	12	−2.64	18	*.008	0.98	0.77
Letter P Fluency	9.5	4.9	12	17.9	6.1	12	−3.39	18	*.002	2.48	1.54
Letter P Violation	0.2	0.6	12	0.2	0.4	12	−0.09	17	.466	0.25	−0.3
Animal Category	10.7	2.4	12	20.6	5.8	12	−5.03	18	*<.001	1.97	2.21
Animal Category Violation	0.5	0.7	12	0.1	0.3	12	1.63	18	*0.06	0.25	0.803
Trail Making A	98.1	33.8	12	28.3	13.1	12	6.09	11.659	*<.001	11.46	2.85
Trail Making B	292.0	19.3	12	77.7	43.4	12	14.26	18	*<.001	15.03	6.56
Stroop Reading (W)	66.3	16.7	12	89.6	9.8	12	−3.81	18	*<.001	6.12	1.75
Stroop Naming Color ^©^	44.8	16.9	12	61.0	9.3	12	−2.66	18	*.008	6.09	1.38
Stroop Color Word (C-W)	46.0	23.1	12	34.5	8.4	12	1.48	11.341	.083	7.77	0.688

*Note:* One- tailed independent t-test. N = 24. *d* Represents Cohen’s *d*.
